# Effects of Packet Loss on Neural Decoding Effectiveness in Wireless Transmission

**DOI:** 10.3390/brainsci15030221

**Published:** 2025-02-20

**Authors:** Jiaqi Zheng, Yuan Li, Liangliang Chen, Fei Wang, Boxuan Gu, Qixiang Sun, Xiang Gao, Fan Zhou

**Affiliations:** 1 College of Biomedical Engineering and Instrument Science, Zhejiang University, Hangzhou 310027, China; 2 College of Computer Science and Technology, Zhejiang University, Hangzhou 310007, China; 3 Binjiang Institute of Zhejiang University, Hangzhou 310053, China

**Keywords:** neural decoding, packet loss, Gilbert–Elliott model

## Abstract

Background: In brain–computer interfaces, neural decoding plays a central role in translating neural signals into meaningful physical actions. These signals are transmitted to processors for decoding via wired or wireless channels; however, they are often subject to data loss, commonly referred to as “packet loss”. Despite their importance, the effects of different types and degrees of packet loss on neural decoding have not yet been comprehensively studied. Understanding these effects is critical for advancing neural signal processing. Methods: This study addresses this gap by constructing four distinct packet loss models that simulate the congestion, distribution, and burst loss scenarios. Using macaque superior arm movement decoding experiments, we analyzed the effects of the aforementioned packet loss types on decoding performance across six parameters (position, velocity, and acceleration in the x and y dimensions). The performance was assessed using the $R^{2}$ metric and statistical comparisons across different loss scenarios. Results: Our results indicate that sudden, consecutive packet loss significantly degraded decoding performance. For the same packet loss probability, burst loss led to the largest decrease in the $R^{2}$ value. Notably, when the packet loss rate reached 10%, the decoding performance for acceleration dropped to 73% of the original $R^{2}$ value. On the other hand, when the packet loss rate was within 2%, the neural signal decoding results across all packet loss models remained largely unaffected. However, as the packet loss rate increased, the impact became more pronounced. These findings highlight the varying degrees to which different packet loss models affect decoding outcomes. Conclusions: This study quantitatively evaluated the relationship between packet loss and neural decoding outcomes, highlighting the differential effects of loss patterns on decoding parameters, and it proposed some methods and devices to solve the problem of packet loss. These findings offer valuable insights for the development of resilient neural signal acquisition and processing systems capable of mitigating the impact of packet loss.

## 1. Introduction

Neural decoding technology plays a crucial role in brain–computer interface (BCI) research. It can be used to analyze the collected neural signals of participants and obtain their intention or movement information, thereby achieving brain control. Neural signal acquisition, as a data source pathway, is crucial for the effectiveness of neural decoding. The transmission media for neural signal acquisition methods can be divided into two types: wired and wireless. Wired transmission can provide a higher number of channels and bandwidth and is currently a commonly used technical method. Wireless transmission is not affected by cable interference and has minimal impact on the free movement of the subject, particularly in experimental research under free-motion conditions, where it plays an irreplaceable role. The behavioral patterns of animals under free movement conditions are central to the study of neural activity. Wireless transmission avoids the constraints of cables on animals and maximizes the naturalness of the experimental environment. For group experiments such as social behavior studies, wireless transmission avoids the risk of cable entanglement and allows subjects to interact more freely. For small or sensitive animals (e.g., mice, birds, etc.), the additional weight of the cable may significantly affect their locomotion, which is avoided by wireless transmission. With the ongoing advancement in nervous system research, lightweight and flexible wireless brain–computer interface systems are increasingly favored by researchers.

In recent decades, neural decoding technology has been used in various applications, such as motion decoding [1,2,3] and language decoding [4,5], with considerable application prospects in the diagnosis, treatment, and the rehabilitation of neurological diseases [6,7,8]. Neural signal decoding algorithms are central to the effectiveness of neural decoding techniques. Most current decoding approaches rely on a Kalman filter or its variants. However, the Kalman filter assumes linear relationships between variables and Gaussian noise; such assumptions often do not hold in practical applications. To overcome these limitations, Makin et al. [9] proposed a novel neural signal decoding algorithm called recurrent exponential-family harmonium (rEFH). The rEFH model demonstrated superior performance compared to the Kalman filter across various experimental conditions, offering a more robust and flexible approach to neural signal decoding.

Packet loss is inevitable during the recording and decoding of neural signal data, whether a wired or wireless transmission mode is used. When the device is correctly configured, the physical reasons for packet loss may include network congestion and line interference [10,11]. Compared to wired transmission, wireless transmission is more susceptible to external interference, and considering factors such as power consumption, size, and transmission distance, the data transmission bandwidth is smaller compared to that of wired transmission. However, the wireless neural signal acquisition and transmission system developed by related neuroscience instrument companies and research teams, such as CerePlex W (Blackrock Neurotech, Salt Lake City, UT, USA), has gradually been promoted in the field of neuroscience research. The results and processing methods related to data packet loss have been mentioned in some studies. Berger et al. [12] used two 96-channel Exilis Headstages (Blackrock Neurotech, USA) to wirelessly record neural signals from monkeys. In their experiment, the best and worst sessions exhibited loss rates of 1.03% and 6.59%, respectively. They believed that an experimental result bias was introduced in trials with high data loss rates. When trials with loss rates above 5% were removed, significant bias was eliminated. Lee et al. [13] developed a wireless recording system that stably transmitted neural data to a server with a packet loss rate of approximately 3%. Packet losses can occur randomly, but most are caused by crosstalk with nearby wireless communication systems. Simeral et al. [14] used four components provided by Blackrock Neurotech to record neural signals from the participants: a pedestal-mounted wireless transmitter, four polarized planar antennas, wireless receiver, and digital hub. The device achieved a minimum packet loss of 0.4166% and a maximum packet loss of 4.69%. Owing to the use of multiple transmitters, a large number of packet losses in one transmitter has minimal impact on the final decoding result. Hansmeyer et al. [15] used 96-channel Exilis Headstages (Blackrock Neurotech, USA) to record neural signals in monkeys. They reinforced the structure with an average packet loss rate of 1.5% compared to previous studies. In current research, the packet loss rate of wireless devices is generally within 7%. Owing to the electromagnetic environment, device placement, and other conditions, packet loss may improve or worsen. However, quantitative research on the effect of packet loss on neural signal processing is lacking, particularly on subsequent neural decoding.

Packet loss during wireless transmission can be categorized into several types according to specific manifestations. Congestion loss occurs when the buffer overflows owing to a burst of data traffic [16,17,18]. This type of packet loss manifests as a loss of large continuous segments of data packets. Distributed loss often occurs in specific situations, such as signal interference and equipment failure. In terms of statistical probability, this type of packet loss is more likely to be an average distribution [19,20,21]. Burst loss is mostly continuous, meaning that several packet losses manifest once a packet loss occurs. Unlike congestion loss, burst loss is probabilistic. Currently, most studies have used the Gilbert–Elliott model (GE) model to investigate burst loss [22,23,24]. Packet loss is widely believed to affect subsequent data processing. However, in the field of neural signal decoding, a quantitative analysis of the decoding results caused by data packet loss is lacking, particularly the impact of different types of packet loss on neural decoding. In this study, we constructed four different types of packet loss models and used macaque superior arm movement decoding experiments [25] to verify the impact of packet loss on the decoding results. The results indicate that different types of packet loss have significant differences in their impact on the decoding results, providing a reference for future studies on packet loss prevention, the impact of packet loss, and packet loss data processing. Additionally, we developed a wireless neural signal acquisition device and proposed a packet loss re-transmission strategy, effectively reducing the packet loss rate.

## 2. Materials and Methods

### 2.1. Signal Acquisition and Processing

The neural data used in this study were sourced from a publicly available dataset provided by the Sabes lab [26]. The data were recorded from both the M1 and S1 areas of a macaque during the performance of a behavioral task, which involved continuous self-paced reaching movements toward targets arranged in an 8 × 8 square grid using the right arm. The recordings were performed using two 96-channel intracranial microelectrode arrays. This study used all 37 sessions of data from monkey Indy, spanning approximately 10 months and encompassing 20,000 reaching movements. The dataset includes the original waveform data of the spikes and their respective occurrence times. This structure aligns with the characteristics of data under wireless transmission, making it suitable for the development of subsequent packet loss models.

The recurrent exponential-family harmonium (rEFH) [9] is a novel filter introduced by Sabes to address the limitations of the Kalman filter algorithm [27], which is widely used in neural decoding. For the aforementioned data, rEFH outperforms some of the most frequently used decoding algorithms. Hence, in this study, rEFH was selected as the neural decoding algorithm to analyze the effects of packet loss. Please refer to reference [25] for the construction of the rEFH.

**Figure 1:**
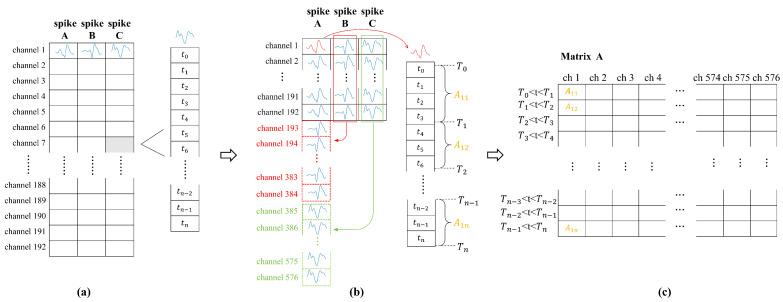
Original data format and reorganized results. (**a**) shows the original matrix derived from a publicly available dataset. In (**b**), data processing is applied, and the primary dimensional transformation treats each spike type as a separate channel, counting the number of spikes within a specified time interval. (**c**) illustrates the matrix upon completing data processing, where each cell corresponds to the number of spikes in the respective channel during a particular time interval.

Before training the neural network, several preprocessing steps were performed on the raw data extracted from the dataset to facilitate subsequent processing. As shown in Figure 1a, the raw neural data are structured in a 192 × 3 cell array, where the 192 rows correspond to 192 channels from the M1 and S1 areas of the monkey, and the three columns represent three distinct spike signal types. Each cell contains a matrix with dimensions *N* × 1, where each entry denotes the time at which a spike signal occurs.

First, we considered neural pathways rather than physical channels, implying that neural signals passing through the same physical channel may originate from different neural pathways. Consequently, the original cell array was transformed into a 576 × 1 cell array, where the 576 rows represent the 576 neural pathways. This is illustrated in Figure 1b.

Subsequently, we constructed a new matrix based on the time information of each cell. By defining a time interval, the number of spikes that occurred within each interval for each neural channel was counted. This resulted in a matrix with dimensions *N* × 576, where the n-th row represents the n-th time interval, and the 576 columns correspond to the 576 neural pathways. Each element in the matrix represents the number of spikes observed in a single neural pathway within a specified time interval. This is illustrated in Figure 1c.

This matrix serves as the foundation for initiating neural network training using both the spike data and location information provided in the dataset. In addition, a packet loss model can be incorporated to simulate the effects of actual packet loss on neural pathways.

In order to control variables and focus on the impact of packet loss on decoding performance, we selected the optimal parameters based on the results presented in reference [25]: bin width (in which spikes are counted and kinematics sampled, 128 ms), training time (the number of seconds of training data, 320), and number of neurons (fraction of all neurons, 100%).

### 2.2. Packet Loss Model

**Figure 2:**
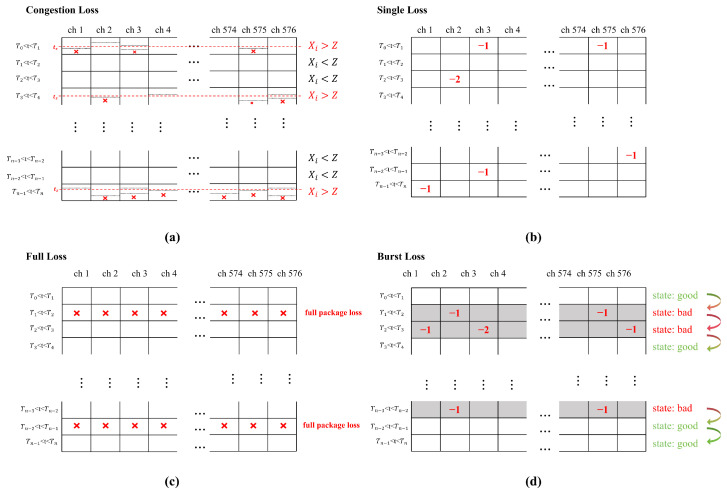
(**a**) Congestion loss model: If the number of packets in a transmission exceeds the bandwidth limit *Z* (red dot line), the spike signals within that transmission are discarded once the cumulative time-ordered signals surpass the bandwidth threshold. (**b**) Single loss model: Randomly discards individual spike signals from the matrix, thereby simulating isolated signal loss within the transmission. (**c**) Full loss model: Considers all spike signals within a specific time interval as a single packet. The model randomly discards entire rows of spike data from the matrix, representing complete loss of information during transmission. (**d**) Burst loss model: Simulates the state of wireless transmission under burst packet loss conditions. No packet loss occurs during favorable transmission states. However, during unfavorable transmission states, entire rows of spike signals in the matrix are randomly dropped, mimicking burst loss events.

Upon completing the preprocessing of the raw data, we obtained a matrix called matrix *A* with dimensions *N* × 576. At this stage, various packet loss models can be applied to the matrix to simulate the effects of actual packet loss on the data. This is shown in Figure 2.

#### 2.2.1. Congestion Loss

Congestion packet loss often occurs when the volume of data being transmitted exceeds the available bandwidth, leading to significant packet loss beyond the network’s capacity. To simulate this type of packet loss, we first calculate the total number of spikes *X* for each row *i* in matrix *A* as follows:

$$X_{i}=\sum_{j=1}^{N}A_{ij}$$

Here, $A_{ij}$ represents the spike count in the *j*-th channel during the *i*-th time interval and *N* indicates the total number of channels. If $X_{ij}$ exceeds the bandwidth *Z*, it corresponds to the number of packets exceeding the bandwidth at a given time interval. All channel spike signals in the interval are sorted from early to late, assuming that the sorting results are $T_{jk}$, where *j* represents the channel and *k* denotes the random ordering of the spike signals for this channel. Using the sorting results, the congestion loss mode can be simulated using the following formula:

$$A_{ij}=\left\{\begin{array}{cc} A_{ij} & \mathrm{if}\;T_{jk}<Z,\\ A_{ij}-1 & \mathrm{if}\;T_{jk}>Z. \end{array}\right.$$

This process is repeated until all the rows have been traversed, resulting in a modified matrix *A*′.

#### 2.2.2. Distributed Loss

According to different data packaging methods, distributed packet loss can be categorized into two types: full packet loss and single packet loss.

The full packet loss model assumes that neural signals are transmitted in discrete complete packet formats. When packet loss occurs in this transmission mode, all neural signal data within the affected packet are lost. Each row represents a complete packet of spiked data in the resulting matrix *A*.

To simulate the full packet loss model within the neural signal matrix *A*, we first calculate the total number of packets to be lost, denoted as *L*, using the following formula:

$$L=\left\lfloor p\times N\right\rfloor$$

where *p* represents the packet loss rate, and *N* is the total number of rows in matrix *A*. Subsequently, we randomly select *L* rows from the matrix using the MATLAB R2024a function *randperm* and set their values to zero, thereby simulating a full packet loss model. The resulting matrix, denoted *A*′, reflects the effect of packet loss with the selected rows set to zero.

Single packet loss assumes that only neural signals are transmitted. To simulate packet loss within the neural signal matrix *A*, we first calculate the total number of packets lost *L* as

$$L=\left\lfloor p\times N\times 576\right\rfloor$$

where *p* denotes the packet loss rate. Subsequently, we randomly select elements $A_{ij}$ within the matrix and decrement their values by one, provided that $A_{ij}>0$. This process is repeated until *L* packets are lost, resulting in a modified matrix, *A*′.

#### 2.2.3. Burst Loss

Burst loss refers to the consecutive loss of multiple packets within a short duration, often caused by network congestion, interference, or environmental disturbances. To simulate this pattern, we used the Gilbert–Elliott (GE) model, which is well suited for capturing the temporal correlation of packet losses. The GE model is a classic channel error model used to characterize the burstiness and temporal correlation of errors, such as packet loss or bit errors, in communication channels. By employing a Markov chain to simulate dynamic state transitions, this model effectively captures the phenomenon of error clustering observed in real-world wireless channels, such as consecutive packet loss in WiFi or cellular networks.

In this study, the GE model is applied to simulate packet loss in the neural signal matrix *A*. This model operates on a two-state Markov process, where each state corresponds to a specific packet transmission condition: the “Good” state (no packet loss) and the “Bad” state (packet loss). Transitions between these states are governed by the following equation:

$${\mathrm{state}}_{t+1}=\left\{\begin{array}{cc} \mathrm{bad} & \mathrm{if}\;{\mathrm{state}}_{t}=\mathrm{good}\;\mathrm{and}\;P_{\mathrm{good}\to \mathrm{bad}}\;\mathrm{is}\;\mathrm{satisfied},\\ \mathrm{good} & \mathrm{if}\;{\mathrm{state}}_{t}=\mathrm{bad}\;\mathrm{and}\;P_{\mathrm{bad}\to \mathrm{good}}\;\mathrm{is}\;\mathrm{satisfied},\\ {\mathrm{state}}_{t} & \mathrm{otherwise}. \end{array}\right.$$

Here, $state_{t}$ represents the state of the transition at time *t*, and $state_{t+1}$ represents the state at a subsequent time step. In the “Bad” state, a portion of the signals in the corresponding rows of matrix *A* are set to zero, simulating packet loss. The resulting matrix *A*′ reflects the impact of the packet loss modeled by the GE process. Assuming that the probability of transitioning from the “Good” state to the “Bad” state is *p* and that the probability of transitioning from the “Bad” state to the “Good” state is *q*, then the overall probability of packet loss is

$$P_{loss}=1-q/\left(p+q\right)$$

Due to various factors influencing packet loss rates in wireless networks, such as network protocols, environmental interference, signal strength, and device performance, it is not feasible to specify a single, fixed value. However, reference ranges can be provided based on typical scenarios and literature. For instance, WiFi networks generally experience packet loss rates of around 1% to 5%, with the possibility of exceeding 10% under strong interference [28]. LoRaWAN typically see packet loss rates ranging from 1% to 10%, which increases with transmission distance [29]. Considering the selection of effective neural signal data in other studies [12,13,14,15], the packet loss rate in this research was set within the 0–10% range. Additionally, the congestion loss model was bandwidth-dependent in this study, meaning packet loss in this model is controlled by adjusting the bandwidth size, with rates ranging from 0 to +∞.

### 2.3. Evaluation Indicator

The rEFH algorithm generates six parameters: position *XY*, speed *XY*, and acceleration *XY*. They represent the reconstruction of the monkey’s motion information at the levels of position, speed, and acceleration. The coefficient of determination $R^{2}$ is a metric commonly used in statistics and regression analyses to measure how well a regression model explains variation in the data. Specifically, $R^{2}$ quantifies the proportion of variance in the observed data that is explained by the predictions of the model. The formula is given as

$$R^{2}:=1-\frac{\langle {\left(x-\hat{x}\right)}^{2}\rangle }{\langle {\left(x-\langle x\rangle \right)}^{2}\rangle }$$

where $\hat{x}$ represents the predicted values of the model, *x* corresponds to the actual observed value, and 〈·〉 denotes the expectation operator (i.e., the mean). The numerator $\langle {\left(x-\hat{x}\right)}^{2}\rangle$ represents the mean squared error (MSE) between the observed and predicted values, and the denominator $\langle {\left(x-\langle x\rangle \right)}^{2}\rangle$ represents the total variance of the observed data with respect to its mean.

The coefficient of determination, $R^{2}$, yields a value between 0 and 1. A value of 1 indicates that the model fully explains the variance in the data, while a value of 0 suggests that the model does not explain any of the variance. In this study, we calculated $R^{2}$ for the six parameters of the rEFH algorithm, comparing the algorithm’s outputs with actual data. This approach allows us to assess the impact of packet loss on neural decoding, where a higher $R^{2}$ value indicates a lesser impact of packet loss.

### 2.4. Statistical Analysis

Parametric statistical tests, specifically one-way analysis of variance (ANOVA), were conducted in this study using Excel. One-way ANOVA was employed to analyze differences between groups under varying packet loss conditions.

## 3. Results

The rEFH algorithm generates six-dimensional outputs, including position (x, y), velocity (x, y), and acceleration (x, y). By comparing the outputs of the algorithm with actual results and calculating the evaluation parameter $R^{2}$, the effect of different packet loss models on the performance of the rEFH algorithm can be rigorously analyzed.

### 3.1. Results of Packet Loss Model Construction

**Figure 3:**
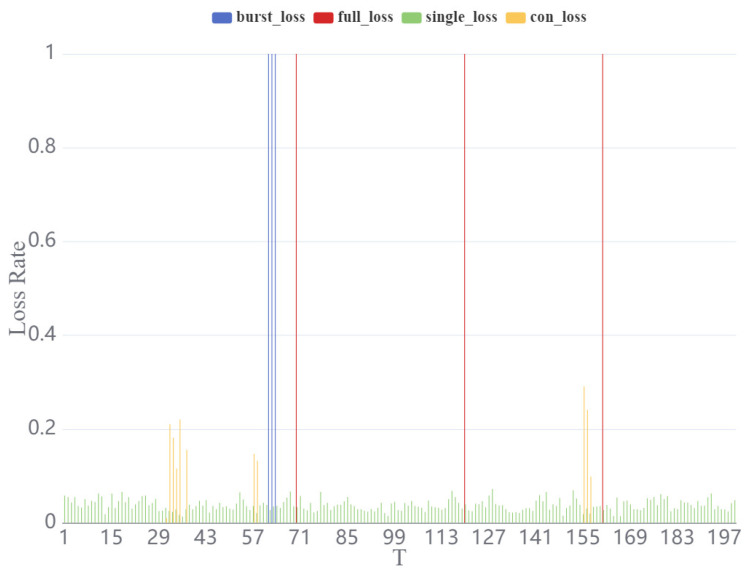
Variation in packet loss rate over time, highlighting the behavior of four distinct packet loss models: burst loss, full loss, single loss, and congestion loss. The packet loss rate is plotted on the vertical axis, while time is represented on the horizontal axis. Each packet loss model is distinguished by a unique color, enabling a clear comparison of their respective trends over time.

The results of the packet loss simulations are shown in Figure 3. The average packet loss rate for each model was maintained at 2%. For the single-loss model, the packet loss rate remained consistent across the time intervals and fluctuated within a narrow range of approximately 2%. In the full-loss model, all packets were dropped at random time intervals, and the packet loss points were scattered more unpredictably. In the congestion loss model, packet loss was concentrated within specific intervals, with loss rates generally remaining below 40% and most variations occurring between 0 and 20%. The burst loss model exhibited a packet loss rate of 100% at random intervals. Unlike the full loss model, these intervals were more continuous, with consecutive time periods where the loss rate remained at 100%.

### 3.2. Comparative Neural Decoding Results Under Different Packet Loss Models

#### 3.2.1. Congestion Loss

**Figure 4:**
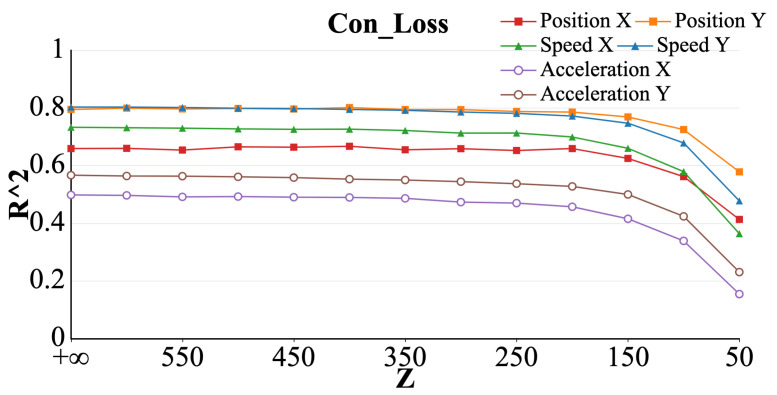
Curve representing the $R^{2}$ value of acceleration (X, Y), velocity (X, Y), and position (X, Y) with decreasing bandwidth constraints under the congestion loss model.

As shown in Figure 4. When the set *Z* was varied to larger values (corresponding to a greater wireless transmission bandwidth), no significant change in the $R^{2}$ values representing the six parameters was found (*p* > 0.05). However, as *Z* gradually decreased toward a critical threshold, the $R^{2}$ values for all six parameters exhibited a noticeable decline. The trends in $R^{2}$ for the different parameters were largely consistent as *Z* varied, with speed and acceleration being more significantly affected by changes in *Z* than the other parameters (*p* < 0.05).

#### 3.2.2. Distributed Loss

**Figure 5:**
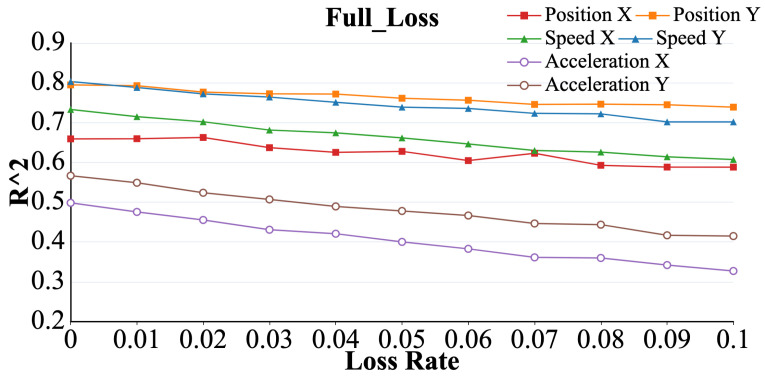
Curve representing the $R^{2}$ value of acceleration (X, Y), velocity (X, Y), and position (X, Y) with increasing packet loss rate under the full packet loss model.

As shown in Figure 5. Under the full loss model, $R^{2}$, which represents the six parameters, exhibited a decreasing trend as the packet loss rate increased (*p* < 0.05). Among these, the $R^{2}$ value associated with acceleration showed the most remarkable change owing to packet loss, while the $R^{2}$ value corresponding to position was least affected.

**Figure 6:**
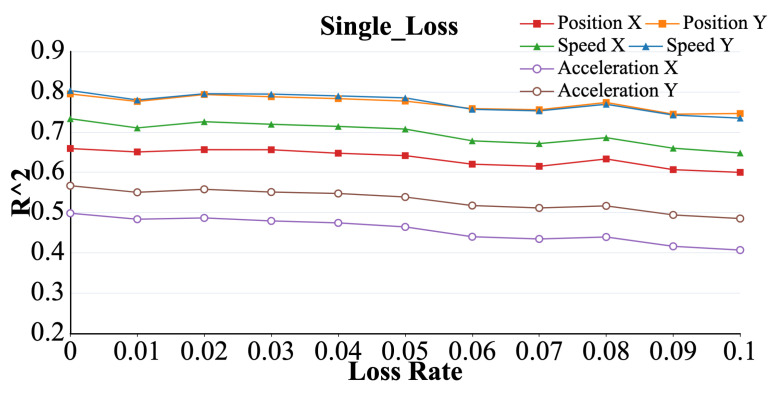
Curve representing the $R^{2}$ value of acceleration (X, Y), velocity (X, Y), and position (X, Y) with increasing packet loss rate under the single loss model.

As shown in Figure 6. Under the single loss model, The $R^{2}$ values representing the six parameters decreased in volatility as the packet loss rate increased. Among these, the $R^{2}$ value for acceleration showed the greatest change in magnitude (*p* < 0.05), whereas the overall trend and magnitude of the changes in the $R^{2}$ values for position and velocity were similar.

#### 3.2.3. Burst Loss

**Figure 7:**
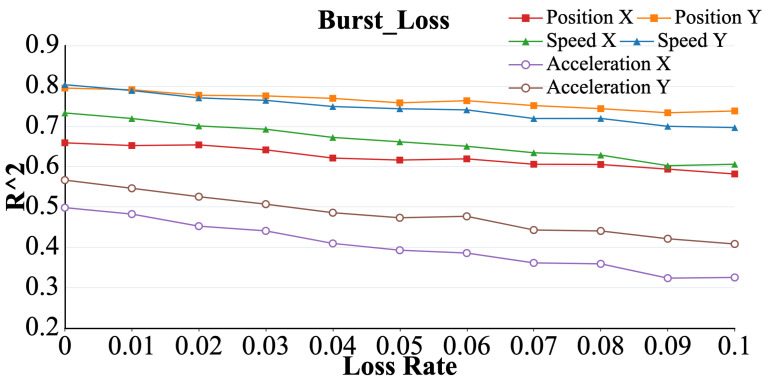
Curve representing the $R^{2}$ value of acceleration (X, Y), velocity (X, Y), and position (X, Y) under the burst loss model with increasing packet loss rate.

As shown in Figure 7. The $R^{2}$ values representing the six parameters gradually decreased as the packet loss rate increased (*p* < 0.05). Among them, $R^{2}$ for acceleration exhibited the most significant change, whereas the $R^{2}$ values for the position and velocity followed similar overall trends and magnitudes.

### 3.3. Performance of Different Decoding Parameters on Packet Loss Data

**Figure 8:**
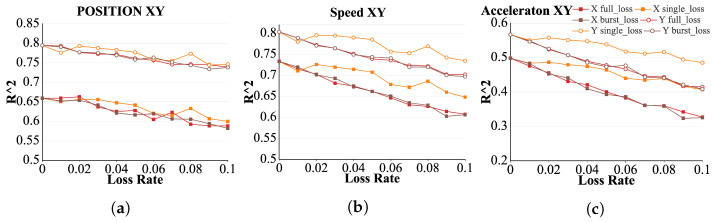
(**a**) Curve of $R^{2}$ values representing position (X, Y) as a function of increasing packet loss rate for different packet loss models. (**b**) Curve of $R^{2}$ values representing velocity (X, Y) as a function of increasing packet loss rate for different packet loss models. (**c**) Curve of $R^{2}$ values representing acceleration (X, Y) as a function of increasing packet loss rate for different packet loss models.

The $R^{2}$ representing the position parameter *XY* exhibited a decreasing trend as the packet loss rate increased across the different packet loss models, as shown in Figure 8a. Under the full loss and burst models, the decline in $R^{2}$ was more gradual with increasing packet loss rate (*p* < 0.05). By contrast, under the single loss model, $R^{2}$ exhibited a fluctuating downward trend, with occasional small increases as the packet loss rate increased. This suggests that the $R^{2}$ for parameter *XY* is the least affected by the packet loss rate under the single loss model and most affected by the burst model. Furthermore, the $R^{2}$ values for parameters *X* and *Y* were similarly affected by various packet loss models, with the primary difference observed in the full loss model. In this model, the $R^{2}$-representing parameter *X* demonstrated a more complex, fluctuating downward trend, whereas the $R^{2}$-representing parameter *Y* followed a steadier downward trajectory.

The $R^{2}$ values representing the velocity parameter *XY* showed an overall decreasing trend as the packet loss rate increased across the different packet loss models. This is illustrated in Figure 8b. The overall decline was similar in both the full and burst loss models, with comparable rates of decrease. Contrastingly, the single loss model exhibited a fluctuating but decreasing trend, with a smaller overall reduction compared with the other packet loss models. In addition, the decreasing trends of the speed parameters *X* and *Y* were largely consistent across the different models.

The $R^{2}$ representing the acceleration parameter generally exhibited a moderate decreasing trend as the packet loss rate increased across different packet loss models. This is illustrated in Figure 8c. The magnitude and trend of the decrease were nearly identical for both the full loss and burst loss models, whereas the single loss model showed a significantly smaller reduction than the other two models. The trends for both the acceleration parameters *X* and *Y* were consistent across all packet loss models.

Among the position, velocity, and acceleration parameters, the $R^{2}$ of the position parameter was the least affected by packet loss, exhibiting the smallest overall decrease. By contrast, the $R^{2}$ of the acceleration parameter was the most affected by packet loss, with the largest overall reduction.

### 3.4. Reconstruction Results

**Figure 9:**
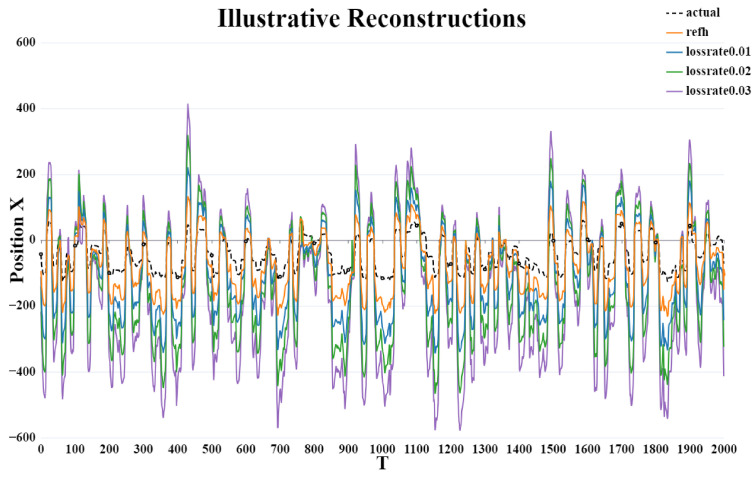
Illustrative reconstructions. Reconstructions of a typical reach (dotted black lines) using the rEFH position decoders with the burst loss model. Only sagittal (x) kinematics (position) are shown, as the lateral (y) reconstructions are similar. These reconstructions correspond to the test data for session Indy2016040702.

As shown in Figure 9. The overall reconstruction results revealed a clear trend: as the packet loss rate increased, the deviation of the position reconstruction in the X direction progressively grew. The reconstruction that most closely aligned with the actual position was obtained using the rEFH algorithm without incorporating any packet loss model. Conversely, the greatest deviation from the actual position occurred when the burst packet loss model, with a packet loss rate of 0.03, was applied. Despite variations in the extent of deviation across different packet loss rates, the general trend in the reconstruction results remained consistent.

## 4. Discussion

In this study, we investigated the effects of various packet loss models on neural decoding outcomes. Specifically, we designed four distinct packet loss models based on different packing methods and wireless transmission characteristics. These models were applied to experimental data involving macaque reach kinematics for testing and analysis. In contrast to previous studies that tended to discard data entirely when faced with significant packet loss, in this study, we adopted a different approach by analyzing and comparing how different types of packet loss affect the decoding performance. This shift in methodology enabled us to explore alternative strategies for managing lost data, offering insights into the impact of different packet loss models on the decoding process. Our findings highlight the importance of understanding the specific characteristics of packet loss, because different types of loss can have varying effects on the accuracy of the neural decoding process. This analysis provides critical guidelines for handling packet loss in future neural signal processing and decoding applications, particularly in wireless systems, where packet loss is inevitable.

### 4.1. Continuous and Burst Packet Loss Have Greater Impact on Decoding

Our analysis shows that the congestion loss model had a minimal impact on decoding position, velocity, and acceleration in both the *X* and *Y* directions. In scenarios with sufficient bandwidth, where ample spike information was available, the decoding performance remained stable, and ideal results can still be achieved despite the packet loss caused by network congestion. For the main experiments in this study, spikes were binned at 128 ms intervals. The single loss model reduced the spike counts in every bin, although the complete loss of an entire bin was exceedingly rare. This ensured that the continuity of neural signals along the timeline was preserved. By contrast, the full loss model, which resulted in the loss of complete data bins, disrupted data continuity. Even when the total number of lost bits was similar, the loss of entire data packets resulted in a poorer decoding performance compared to the single loss model. A key insight derived from these results is the importance of designing data packaging strategies that minimize the size of the data units being transmitted. The complete loss of packets can be avoided by reducing the packet size, thereby preserving the continuity of the neural signals and improving decoding performance. However, this approach has certain trade-offs. Smaller packets require additional metadata (such as headers, tails, and checksum) for transmission, which reduce the overall efficiency and increase bandwidth usage. Therefore, it is crucial to strike a balance between the packaging size and transmission efficiency.

The GE loss model approximates real-world conditions more closely and demonstrates how disruptions in data continuity significantly affect decoding performance. In the GE model, transitions between good and bad states affect the integrity of the transmitted data. To mitigate the impact of packet loss, it is essential to decrease the transition probability from a good state to a bad state while simultaneously increasing the probability of returning from a bad state to a good state. The primary strategy is to enhance transmission quality to reduce the likelihood of entering a bad state. However, if a transition to a bad state occurs, minimizing the packet loss and rapidly returning to the good state are critical. This can be achieved using various techniques, including automatic transmission requests (ARQs) [30], which request to resend lost packets; forward error correction (FEC) [31], which adds redundancy to the data stream to enable the recovery of lost packets without re-transmission; and transmission rate adjustment, wherein the transmission rate is dynamically adjusted to adapt to the network conditions and reduce packet loss. These strategies can be applied to reduce the detrimental effects of packet loss and maintain higher neural decoding accuracy, particularly in systems where data continuity is crucial.

### 4.2. Decoding with Higher Data Continuity Requirements Is More Affected by Packet Loss

In this decoding experiment, the spike counts were a linear function of these variables as well as the six kinematic states. Owing to the sparsity of the processed spike count matrix and the relatively slow changes in position compared to velocity and acceleration, a small amount of packet loss had minimal impact on position decoding.

However, packet loss or signal discontinuities can directly affect the accuracy of these estimates because velocity and acceleration are derivatives of position and depend on dynamic discharge patterns in neural signals over short time intervals. This is because the derivatives amplify fluctuations or inconsistencies in the underlying signal. In addition, the recursive nature of the decoding algorithm can exacerbate errors in velocity and acceleration estimation, accumulating prediction errors. These accumulated errors further degrade the decoding performance, particularly velocity and acceleration, which are highly sensitive to small temporal changes.

Consequently, neural decoding algorithms that demand high data continuity and integrity are particularly sensitive to packet loss. By contrast, algorithms based on sparse matrices or averaged spike rate data may exhibit a slightly better tolerance to packet loss because of the smoother or less dynamic nature of the data being processed.

### 4.3. Relationship Between Packet Loss Rate and Decoding Result

The accuracy requirements for decoding results varied based on the evaluation criteria and specific application scenarios. For example, in a rat compression bar experiment [32], the goal was to determine whether the bar had been pressed, that is, it was a binary classification task. In such cases, a high decoding accuracy may not be essential, and the system can tolerate a certain amount of packet loss without significantly affecting performance.

Contrastingly, for trajectory prediction tasks, such as the experiment presented in this study, the ideal scenario is for the decoded motion trajectory to perfectly match the actual trajectory, an outcome where $R^{2}$ is one. However, no existing decoding algorithms can achieve such high-performance metrics. Thus, in regression tasks such as this, it is crucial to minimize the packet loss to maximize decoding accuracy.

In this study, we found that a packet loss of less than 2% ensured that the difference in $R^{2}$ values between the predicted and true trajectories was within 0.05. Additionally, the impact of the packet loss on the position decoding was found to be slightly smaller than that of velocity and acceleration.

### 4.4. Optimization and Validation of Wireless Neural Signal Transmission

Currently, there are many kinds of neural signal decoding algorithms, and it is obviously not feasible to conduct a comprehensive packet loss model test for each algorithm. However, if the packet loss rate of neural signals can be significantly reduced by effective measures before decoding, it will lay an important foundation for improving the accuracy of various decoding algorithms. Such optimization not only helps to improve the reliability of decoding results but also provides more robust data support for the applicability of different decoding methods.

**Figure 10:**
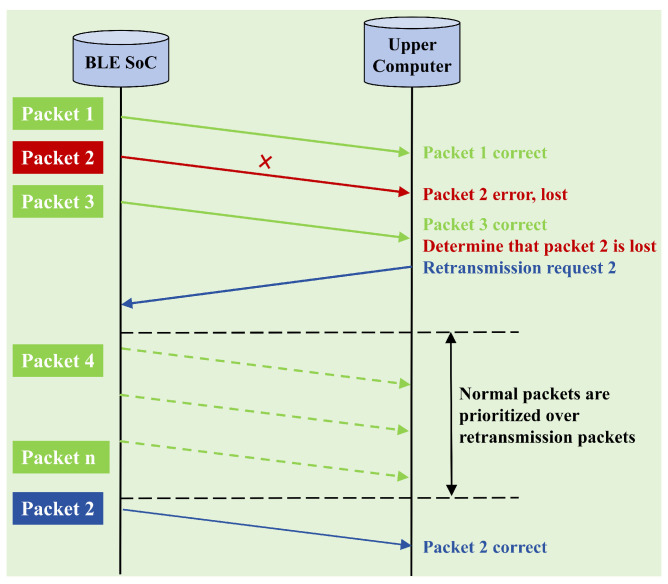
Diagram of packet loss re-transmission mechanism.

In order to optimize the packet loss problem during wireless transmission of neural signals, we designed and implemented a packet loss re-transmission mechanism. As shown in Figure 10. This mechanism was applicable to the data interaction between the wireless neural signal acquisition device and the host computer. Specifically, the host computer detected the continuity of the packet by parsing the identification (tag) in the received packet and recorded the relevant information of the lost packet. When packet losses were detected, the host computer sent a re-transmission request to the neural signal acquisition device. If the bandwidth condition allowed, the acquisition device performed a re-transmission operation for the lost packet. This mechanism can significantly reduced the packet loss rate during wireless transmission and provided more reliable data guarantee for the accuracy of neural signal decoding.

At the same time, we developed a wireless neural signal acquisition device, which is capable of acquiring 64-channel neural signals and wirelessly transmitting the data to the host computer via Bluetooth. Based on this device, we conducted functional verification and performance evaluation of the packet loss re-transmission mechanism to further demonstrate its effectiveness in practical application scenarios.

**Figure 11:**
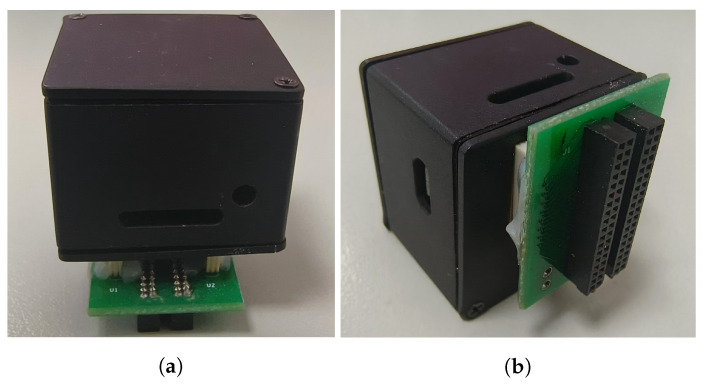
Wireless neural signal acquisition device. (**a**) Front view of self-designed wireless nerve acquisition device, (**b**) Side view of self-designed wireless nerve acquisition device.

**Figure 12:**
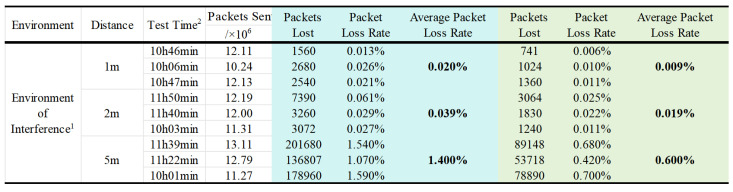
Actual test results of packet loss re-transmission. ^1^ Interference environment with more than 20 detectable Bluetooth devices, more than 10 WiFi devices, and more than 10 active people. ^2^ The device was charged for a long and full test period. The blue background represents the packet loss of the device without packet re-transmission enabled, while the green background indicates the packet loss with packet re-transmission enabled.

We evaluated the effectiveness of the packet loss re-transmission mechanism using a self-designed wireless neural acquisition device. As shown in Figure 11. In an interference-prone environment, long-duration transmission tests were conducted at distances of 1 m, 2 m, and 5 m. During these tests, the total number of packets sent was recorded, and the packet loss rate, along with its average value, was calculated. As shown in Figure 12. The results demonstrated a significant reduction in packet loss rates when the re-transmission mechanism was employed, with the packet loss rate reduced to approximately half of the original value. The reduction in packet loss rate was also instrumental in enhancing the accuracy and reliability of neural signal decoding.

We compared the proposed packet loss re-transmission method and its test results with other strategies and packet loss outcomes related to wireless neural signal acquisition from the other works, as shown in Table 1. The strategy adopted in some works is to discard or assign zero processing to lost data, such as [12,13]. This strategy may be effective when the packet loss rate is low. However, as the packet loss rate increases, it directly impacts the decoding process. Other strategies focus more on improving wireless signal quality, such as using an EMI-shielded room, multi-antenna, stable head position, etc. These strategies not only increase costs but also limit the animals’ movement space, making it difficult to conduct free-moving experiments. Our method, from the perspective of transmission strategy, can control the packet loss rate under limited device conditions, making it a more cost-effective and versatile solution.

### 4.5. Limitations and Future Works

This study also has several limitations.

First, given the high precision required for the kinematic decoding, the analysis was limited to a specific kinematic experimental paradigm. However, the packet loss model developed in this study can also be applied to other forms of neural decoding, broadening its potential for use in future studies.

Second, the evaluation metrics for assessing the impact of packet loss on the decoding results lack universality. Currently, the impact is gauged through performance indicators such as decoding accuracy or trajectory similarity, which vary across different decoding algorithms. This variation complicates the subsequent processing of packet loss data because each algorithm requires tailored evaluation criteria. Developing more universal metrics or models could streamline the data filtering and handling processes in future studies.

Finally, although this study analyzes the issues in the wireless signal transmission process, experiments were conducted only on rEFH and macaque data, with in vivo experiments not yet performed. Future work will involve using the wireless devices developed in this study, which integrate packet loss re-transmission, for in vivo implementation on multiple subjects and species, to validate the effectiveness of the proposed method.

## 5. Conclusions

This study investigated the impact of packet loss on neural signal decoding by constructing four distinct packet loss models. The results demonstrate that packet loss significantly affects decoding accuracy, particularly at high packet loss rates. At the same packet loss probability, successive bursts of packet loss resulted in a more pronounced degradation in the decoding performance, underscoring the importance of preventive strategies, such as packet re-transmission. By contrast, occasional packet loss had a relatively minor impact on the decoding results within certain thresholds. The rEFH model selected for this study exhibited superior robustness and accuracy in neural signal decoding compared to traditional methods such as the Kalman filter, highlighting its practical advantages in real-world applications. The insights and models presented in this study provide valuable references for addressing the packet loss problem in future neural signal acquisition, particularly in wireless transmission environments where packet loss is more prevalent. Our findings highlight the importance of designing robust transmission strategies to mitigate the impact of packet loss on neural decoding accuracy, offering a foundation for future research in resilient BCI systems.

## Figures and Tables

**Table 1:** Summary of packet loss rate and strategies in wireless neural signal acquisition.

Reference	Strategy	Packet Loss Rate
Berger et al. [12]	Use the data with a loss rate of less than 5%.	1.03% to 6.59%
Lee et al. [13]	1. The amplitude at the point of packet loss is recorded as zero; 2. Suppress crosstalk by EMI-shielded room.	3%
Simeral et al. [14]	Use four antennas and two transmitters.	0.4166% to 4.69%
Hansmeyer et al. [15]	Stable head position and strategic placement of the receiving antennas	1.50%
This paper	Packet loss re-transmission mechanism	0.60%

## Data Availability

The data presented in this study are available in zenodo at https://doi.org/10.5281/zenodo.3854034.
